# Quantification of liver fibrosis in chronic hepatitis B virus infection

**Published:** 2015

**Authors:** CF Jieanu, BS Ungureanu, DL Săndulescu, IA Gheonea, DR Tudorașcu, ME Ciurea, VL Purcărea

**Affiliations:** * Research Center of Gastroenterology and Hepatology, University of Medicine and Pharmacy, Craiova, Romania; ** ”Carol Davila” University of Medicine and Pharmacy, Bucharest, Romania

**Keywords:** HBV, non-invasive methods, fibrosis, end-stage liver disease

## Abstract

Chronic hepatitis B virus infection (HBV) is considered a global public issue with more than 78.000 people per year dying of its evolution. With liver transplantation as the only viable therapeutic option but only in end-stage disease, hepatitis B progression may generally be influenced by various factors. Assessing fibrosis stage plays an important part in future decisions on the patients’ wealth with available antiviral agents capable of preventing fibrosis passing to an end-stage liver disease. Several methods have been taken into consideration as an alternative for HBV quantification status, such as imaging techniques and serum based biomarkers. Magnetic resonance imaging, ultrasound, and elastography are considered non-invasive imaging techniques frequently used to quantify disease progression as well as patients future prognostic. Consequently, both direct and indirect biomarkers have been studied for differentiating between fibrosis stages. This paper reviews the current standings in HBV non-invasive liver fibrosis quantification, presenting the prognostic factors and available assessment procedures that might eventually replace liver biopsy.

## Introduction

Chronic hepatitis B virus infection (HBV) is considered a global public issue with more than 78.000 people per year dying of its evolution [**1**]. With liver transplantation as the only viable therapeutic option but only in end-stage disease [**2**], hepatitis B progression may generally be influenced by various factors. No doubt liver fibrosis progression to cirrhosis enhances the risk of hepatocellular carcinoma (HCC) [**3**,**4**] therefore, a good quantification and prediction of substantial patient developments is necessary. Assessing fibrosis stage plays an important part in future decisions on the patients’ wealth. Antiviral agents have the capability of potentially preventing fibrosis passing to an end-stage liver disease, by maintaining undetectable levels of HBV DNA [**5**].

Traditionally, the gold standard for staging fibrosis is considered the pathologic interpretation after liver biopsy, but with its limitations, as it has several flaws and possible complications [**6**,**7**].

Several methods have been taken into consideration as an alternative for HBV quantification status, such as imaging techniques and serum based biomarkers. Magnetic resonance imaging, ultrasound, and elastography are considered non-invasive imaging techniques frequently used to quantify disease progression as well as patients future prognostic. Consequently, both direct and indirect biomarkers have been studied for differentiating between fibrosis stages [**8**].

This paper reviews the current standings in HBV non-invasive liver fibrosis quantification, presenting the prognostic factors and available assessment procedures that might eventually replace liver biopsy.

## From Fibrosis to end-stage disease

HBV fibrogenic activity may be described as a chronic inflammation with a clinical progression to cirrhosis and HCC in most of the patients. Viral activity produces constant liver damage on the immune system, offering in response continuous tissue repairing but in a disorganized matter. Additionally, a viral X protein implication in cellular DNA activity has been studied with potential angiogenic, fibrogenic, and oncogenic effects [**9**]. Apparently, HBV X occupies an important role in virus replication by affecting human hepatic stellate cells activation.

Both cirrhosis and HCC as evolutionary steps of fibrosis are related to high morbidity and mortality rates [**10**,**11**]. D’Amico et al. [**11**] published a review on a large cohort of patients, pointing out that the mortality risk increases after every decompensation, varices appearance, variceal bleeding and ascites. Slowing down the progression process is one of the main objectives of HBV treatment. With over 350.000 HCC deaths worldwide every year [**12**], some of them are related to untreated patients, with an incidence of 0.3%-0.6% in non cirrhotic patients and 2.2 – 3.7% in compensated cirrhotic patients [**13**,**14**].

After exposure to HBV infection, a proper management is definitely necessary. With clinical outcomes from inactive carrier state to end-stage liver disease, a close monitoring with a periodic evaluation are in need to prevent future complications. Both radiologic and serum biomarkers may yield valuable information on how to asses clinical disease evolution and associate available therapies.

Several risk factors have to be taken into consideration if associated to HBV, as they may accelerate the course of liver fibrosis progression such as male gender, older age, alcohol consumption, elevated alanine aminotransferase (ALT) levels, high HBV DNA level and nonetheless associated hepatitis C or D virus and also human immunodeficiency virus (HIV) [**15**]. Also HBeAg presence is known to potentially advance fibrosis [**16**]. A recent study on 377 patients with both HbeAg positive and negative focused on predicting fibrosis with HBV genotype precore and core variants. Risk factors were classified before patients underwent liver biopsy. Age, ALT, HBV-DNA, and HBV variants were considered strong independent factors as they could predict fibrosis on a METAVIR F ≥2 stage, independent of HBeAg status [**17**]. 

HDV infection is known to offer a grim prognostic in chronic liver disease evolution. While always associated with HBV infection, it can present as an acute co-infection in treatment-naive patients as well as a superinfection in the pre-existing disease. Chronic infection alters the natural history of fibrosis progression with a more rapid development to consecutive cirrhosis, a decompensation status, or HCC, than singular HBV infection [**18**].

Although HIV infection induces a liver disease such as hepatitis, endothelial necrosis, granulomatosis by its own action, it also has the capability of fostering HBV infection. Co-infection status hastens and alters HBV hepatopathogenesis process [**19**,**20**]. With severe decrease in Hbe antigen clearance with almost five times, higher levels of HBV replication are encountered [**21**,**22**]. As the immune system regresses, even seroconverted patience to surface hepatitis B antigen HbsAb develop a chance in reverse-seroconversion and therefore reactivating the infection [**23**,**24**]. The complexity of both co-existing viruses seems to progress more rapidly to HCC, even though cirrhosis is the main evolutionary status of HBV [**25**,**26**].

## Fibrosis Quantification

Borderline diagnosis between HBV and cirrhosis is actually based on fibrosis progression correlated with additional clinical settings. Evaluating HBV status is extremely important for a proper framing and deciding a specific treatment. Although liver biopsy has been considered the gold standard for assessing fibrosis, there are some drawbacks regarding the use of this method. First of all, the biopsy specimens may vary depending on the extract location [**27**]. Thus, a laparoscopic study on 124 patients, on right and left lobe biopsies revealed that almost 14.5% of the patients were staged differently with F3 in one lobe and F4 in another [**27**]. Also, fibrosis evaluation method may be improved if several pathologists would read the specific specimens, a general process which is not cost and time effective for clinical practice [**28**]. On the other hand, variable lengths biopsies may give different results [**6**]. Control quality biopsy study on CHC patients, showed that only 31% of the taken specimens were “adequate” located at least 15 mm and ≥ 5 portal tracts and that only 14% of them were considered “ideal” at ≥ 25 mm [**6**]. Complications are not frequent; most of them are due to hypotension, post-procedural pain, and hemoperitoneum. A large retrospective study of over 68.000 biopsies found a mortality rate of 0.01% [**29**], while another review on 1000 patients delivered a complication rate of 5.9% [**30**].

**Transient Elastography (TE)**

Specific end-to-end non-invasive imagistic methods are now more frequently used for the assessment of liver fibrosis. Transient Elastography – FibroScan (Echosens, Paris, France) is one of the world-used methods in liver fibrosis quantification, even in HBV and CHC [**31**]. With the help of a transducer, mounted on the axis of a vibrator at the end of the US probe, 50 MHz pressure waves are directed on the selected liver tissue. The result consists of painless vibrations that propagate a “shear wave”, representing the velocity of the wave being sent back to the ultrasound. The shear wave is tracked, correlated, and expressed in kilopascals by the device, thus estimating liver fibrosis [**8**]. 

So far, this technique has proven useful in assessing fibrosis [**32**,**33**], identifying cirrhosis complications such as portal hypertension [**34**] and it also has been studied in post-transplant situations in HCV patients [**35**]. Several measurements are necessary for a proper evaluation. Even so, evaluating results has proven difficult since some limitations were encountered. The patient must hold his breath during the procedure to minimize errors [**36**], narrow intercostal space might increase false-positive results [**37**], while fasting conditions are also necessary [**38**-**40**]. An important aspect is the fact that FibroScan cannot be performed on patients with ascites [**41**].

On HBV patients, a meta-analysis correlated the FibroScan values with the METAVIR score. F2 was related to a value 7.9 kPA, while F4 to 11.7 kPa with a sensitivity of 0.859 respectively 0.929 [**42**]. However, some studies showed that results in HBV might be influenced by the aminotransferases flares encountered in patients affected [**43**]. To forecome obesity encountered errors, a 3rd generation device, Fibrotouch (Wuxi Hisky Medical Technology Co Ltd, Beijing, China) is available with potential new measurement depths using a new dynamic probe [**44**].

This procedure has been endorsed by the European Association for the study of the Liver in management of viral hepatitis because of its non-invasiveness, accuracy, and capability of differentiating between absence, mild or advanced fibrosis and cirrhosis [**45**].

**Real Time Elastography **

**Fig. 1:**
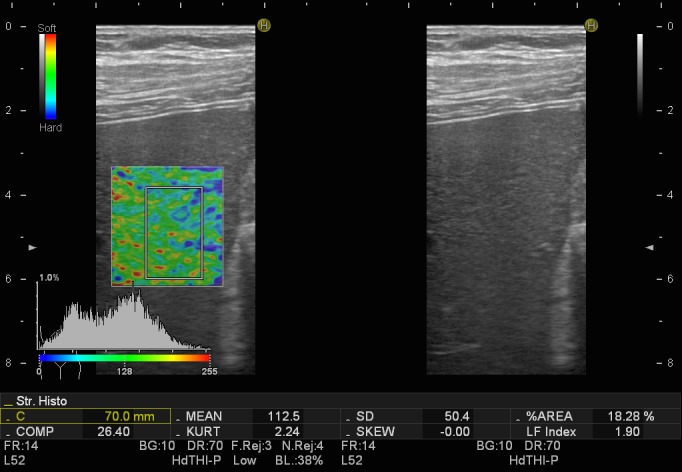
Transabdominal real-time elastography in a patient with liver steatosis (soft appearance of liver tissue)

**Fig. 2:**
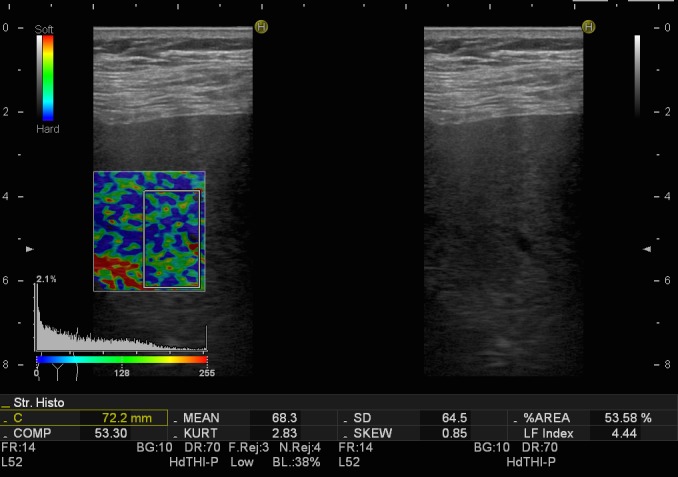
Transabdominal real-time elastography in a patient with HVB (hard appearance of liver tissue)

Real time elastography (RTE) is also a useful and promising technique in measuring liver stiffness, which evaluates a shear wave through the liver, while capturing echo signals in real time. Pressure made on the liver tissue translates tissue elasticity as color-related (red - soft tissue, green - intermediate hardness tissue, blue - hard tissue), therefore correlating the region of interest with specific consistency of the desired area (**Fig. 1**,**2**).

**Fig. 3:**
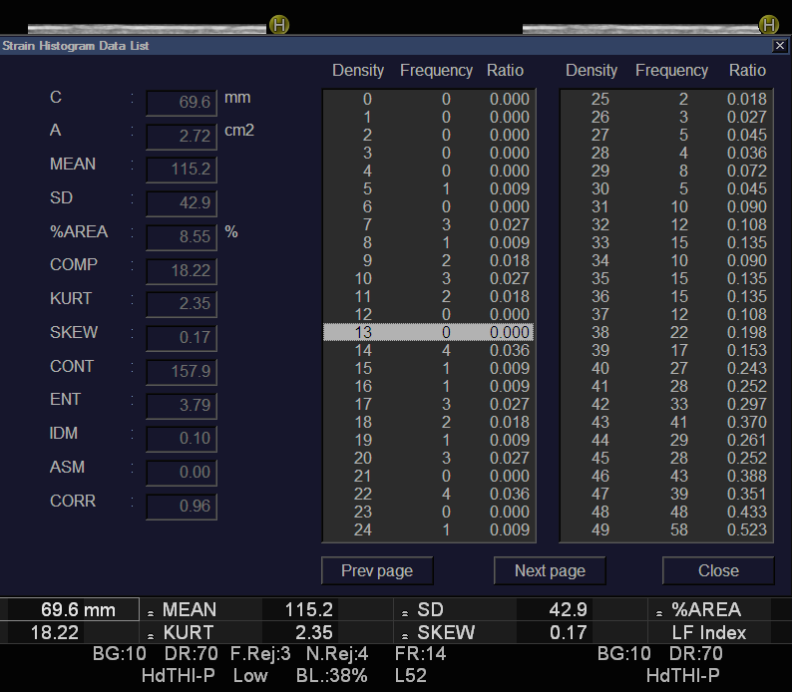
Elastic parameters calculated by an ultrasound system in order to characterize liver stiffness

RTE seems to overcome some of the drawbacks of transient elastography or ARFI procedures such as patient’s tissue motility, obesity, or patients with very stiff tissue. Most of the studies that quantify fibrosis with RTE are using a semi quantitative technology that analyzes 11 elastic parameters used to characterize liver stiffness (**Fig. 3**). 

Xie et al. [**46**] evaluated patients with different fibrosis degrees while calculating elastic strain ratios with a good sensitivity for substantial fibrosis and cirrhosis of 77.8% and 50.0% and specificity of 80.0%, respectively 96.7%. This study assessed liver fibrosis with blood parameters, liver biopsy, and RTE in a specific region of interest with a 3 to 4 pressure measurement on a 0 to 6 scale. When comparing the results of RT-E with the histologic findings, a high correlation between elastic strain ratios and increasing fibrosis stages was noted. Also after blood parameters and Forns indexes analysis, the AUC curves showed that RT-E might be more accurate.

**Acoustic Radiation Force Impulse (ARFI)**

ARFI liver stiffness measurement is performed with a Siemens Acuson S2000 Virtual TouchTM US system (Siemens AG, Erlangen, Germany) and consists of an acoustic “push” pulse directed to a region of interest where the shear wave will be measured [**40**]. There is a recommendation of 10 established measurements with a median value calculation in order to obtain a close to reality value. Some studies, as well as the manufacturer suggest that technical parameters IQR <30% and SR ≥60% increase the method’s accuracy [**47**,**48**]. 

While using this method, as well as in FibroScan testing, fasting is necessary for avoiding false results [**49**,**50**]. Also heart failure and elevated aminotransferase levels should be eluded [**51**,**52**]. A Romanian study on HBV and CHC patients showed concordant values with pathological liver fibrosis [**53**]. 

**Magnetic Resonance Elastography**

Nowadays, MRE is considered one of the most reliable methods for the assessment of liver fibrosis. A transducer is placed under the patient’s thorax and while performing MRI imaging, viscosity maps and shear wave elasticity maps reproduce the liver consistency over a larger area than other available methods [**54**]. A study on 141 patients with chronic liver disease compared the efficacy of TE and MRE for both cirrhosis and fibrosis with clearly higher accuracy in favor of MRE [**54**]. This procedure is capable of distinguishing between moderate or high levels of fibrosis ≥ F2 [**55**] with an increased success rate of 94% and the AUROCs of 0.994 for ≥ F2, 0.985 for ≥ F3 and 0.998 for ≥ F4 [**56**]. 

However, MRE has a major limitation because of its availability, expensive equipment, and professional expertise [**57**]. Also the long time of exposure for each patient could actually be a flaw of the technique. 

## Serological Markers

Liver fibrosis assessment also requires periodic biological sampling. Serum biomarkers are classified in two components direct and indirect in evaluating fibrosis status. While the direct markers reflect the pathophysiology of liver fibrogenesis, representing the extracellular matrix components, the other class follows the consequence of liver damage with routine laboratory analysis [**58**]. For a better assessment, several scores combining these biomarkers have been proposed. The APRI-Test was used in a meta-analysis of 18 studies [**59**]. Using the formula AST/ upper limit of normal *100/ platelet count an estimation of fibrosis is possible as portal hypertension signs result in a decline of platelet count. The test had a specificity of 94% in identifying cirrhosis with a pooled AUROC of 0.84. and a specificity of 55% for the fibrosis diagnosis.

Fibrotest is a patented formula (Biopredictive, Paris, France) which involves several parameters: total bilirubin, haptoglobin, gamma glutamyl transpeptidase, a2-microglobulin apolipoprotein-A, age and gender, with the results correlated with the METAVIR score [**60**]. This evaluation is the most used indirect marker showing excellent results in a meta-analysis in CHC [**61**]. However, it has a lower success rate when determining significant fibrosis [**62**].

Fibroindex is also based on markers such as AST, GGT, and platelet count. It was taken into consideration on a CHC study on 360 patients with encouraging results, with AUROC of 0.82 for significant fibrosis [**63**].

Direct biomarkers targeting the direct pathophysiology of the fibrotic process have been studied. Hyaluronic acid deposited in the extracellular matrix may interfere with liver fibrosis since it is degraded by hepatic endothelial cells [**8**]. The other two biomarkers following collagen distribution have been proposed. TIMP-1 (tissue inhibitors of metalloproteinase) [**64**] and PIIINP (amino terminal of serum procollagen III peptide) [**65**] have been correlated with fibrosis and cirrhosis. 

## Conclusions

Although liver biopsy is the reference standard in identifying fibrosis and evolution to an end-stage disease, the researcher’s and the clinician’s attention has been focused on several imagistic methods and biological markers to potentially overcome the biopsy’s flaws. Even their correlation so far has not brought a universal new accepted standard for the evaluation of fibrosis. With good results in excluding HBV fibrosis or cirrhosis, these methods have surely replaced biopsy in many cases. Without a doubt, evaluating and classifying fibrosis at an early stage represents an important factor for the prediction of evolution to cirrhosis and CHC in HBV patients. However, future randomized and controlled studies of liver fibrotic pathologic status are necessary to identify the perfect non-invasive method of quantifying liver fibrosis in HBV.

**Acknowledgments**

This paper was published under the frame of European Social Found, Human Resources Development Operational Programme 2007–2013, project No. POSDRU/159/1.5/133377.
